# Investigating the capabilities of large vision language models in dog emotion recognition

**DOI:** 10.1038/s41598-025-25199-7

**Published:** 2025-11-21

**Authors:** George Martvel, Anna Zamansky, Ilan Shimshoni, Annika Bremhorst

**Affiliations:** 1 University of Haifa, Haifa, Israel; 2 University of Bern, Bern, Switzerland

**Keywords:** Computational models, Machine learning

## Abstract

Identifying emotional states in animals is a key challenge in behavioural science and a prerequisite for developing reliable welfare assessments, ethical frameworks, and robust human–animal communication models. Recently, large vision-language models (LVLMs) such as GPT-4o, Gemini, and LLaVA have shown promise in general image understanding tasks, and are beginning to be applied for emotion recognition in animals. In this study, we critically evaluated the ability of state-of-the-art LVLMs to classify emotional states in dogs using a zero-shot approach. We assessed model performance on two datasets: (1) the Dog Emotions (DE) dataset, consisting of web-sourced images with layperson-generated emotion labels, and (2) the Labrador Retriever cropped-face (LRc) dataset, which stems from a rigorously controlled experimental study where emotional states were systematically elicited in dogs and defined based on the experimental context in canine emotion research. Our results revealed that while LVLMs showed moderate classification accuracy on DE, performance is likely driven by superficial correlations, such as background context and breed morphology. When evaluated on LRc, where emotional states are experimentally induced and backgrounds are minimal, performance dropped to near-chance levels, indicating limited ability to generalise based on biologically relevant cues. Background manipulation experiments further confirmed that models relied heavily on contextual features. Prompt variation and system-level instructions slightly improved response rates but did not enhance classification accuracy. These findings highlight significant limitations in the current application of LVLMs to non-human species and raise ethical and epistemological concerns regarding potential anthropocentric biases embedded in their training data. We advocate for species-sensitive AI approaches grounded in validated behavioural science, emphasising the need for high-quality, preferably experimentally-based multimodal datasets and more transparent validation. Our study underscores both the potential and the risks of using general-purpose AI to infer internal states in animals and calls for rigorous, interdisciplinary development of animal-centred computational approaches.

## Introduction

Accurately identifying animal emotions is crucial for improving scientific understanding, promoting animal welfare, and ensuring safe and empathetic interactions between humans and animals, both in research and everyday life. Emotions can profoundly influence behavioural responses^1,2^, yet non-verbal species, like all non-human animals, cannot explicitly communicate their internal states. Consequently, we must rely on indirect indicators such as facial expressions, body movements, and behavioural tendencies to infer emotions an animal is likely to experience^3^.

Scientific efforts have made considerable progress in identifying such behavioural emotion indicators, particularly in companion species such as dogs. Among these, facial expressions have been systematically studied as potential non-invasive indicators of canine affective states^4–6^. The Dog Facial Action Coding System (DogFACS)^7^ provides an objective, morphology-independent method for quantifying facial movements, thereby enhancing standardisation and reproducibility in canine facial analysis. DogFACS has been used in multiple studies to identify facial expressions associated with emotional states in dogs^4,6,8,9^. However, relying on isolated behaviours (e.g. a single facial expression such as a specific ear movement) may not be sufficient to reach high emotion classification accuracy, at least when using human-based manual annotation^5^. Given the inherently multimodal nature of emotional expressions, integrating information from diverse sources such as facial expressions, body posture, behavioural tendencies, arousal level, and situational context may help improve the accuracy of emotion detection^5,10^. Nonetheless, establishing reliable behavioural emotion indicators remains a complex challenge. While DogFACS offers a systematic and relatively objective framework for measuring facial movements, its practical application still relies on human coders, requiring extensive training and certification. As a result, it is time-consuming and remains susceptible to human error and bias^11^.

Given these complexities, there is increasing interest in artificial intelligence (AI) models to automate behaviour tracking and emotion recognition in dogs. Supervised deep-learning models trained on experimentally controlled datasets, where specific emotional states are carefully experimentally induced, have demonstrated high classification accuracy for states such as positive anticipation or frustration in dogs^12^. However, while controlled datasets offer high-quality labels for model training, such datasets are rare, and this scarcity limits scalability.

**Fig. 1:**
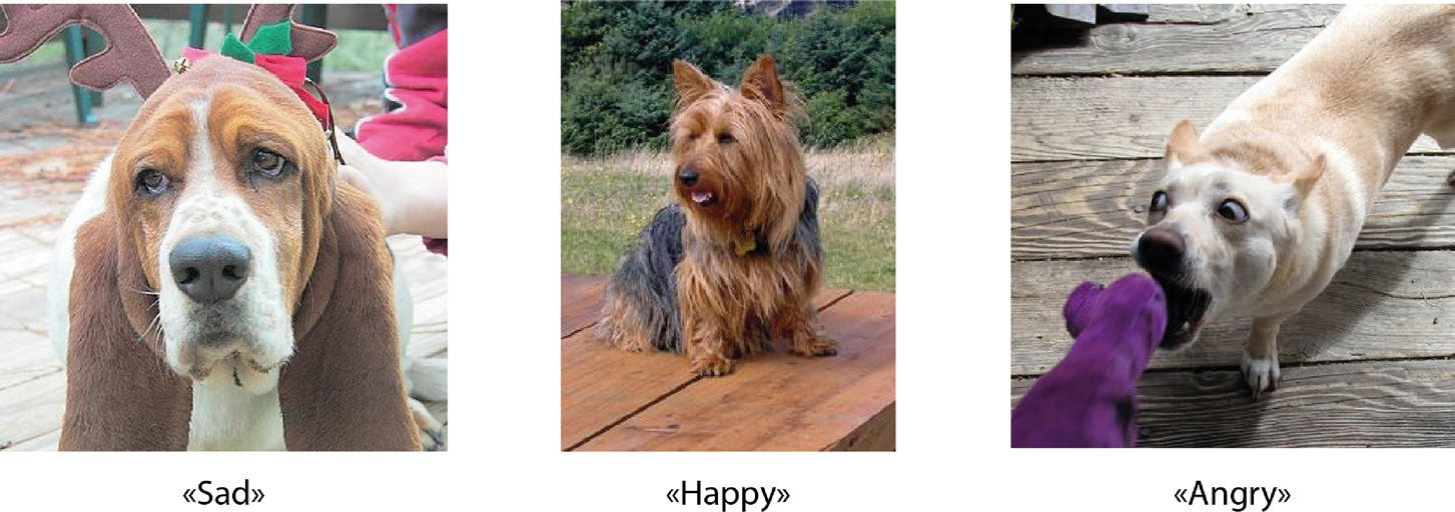
Images from the Dog Emotion dataset^15^ with the corresponding labels.

This has led some researchers to explore internet-based image datasets^13,14^, attempting to classify emotional states such as happiness, anger, or relaxation in dogs. Figure 1 shows example images from the Dog Emotion dataset^15^ with their corresponding labels. These datasets are typically sourced from uncontrolled contexts and often lack critical contextual information, such as the behavioural sequence, environmental setting, or emotion-competent stimuli present in the setting, which can be crucial for accurately interpreting affective states^4,10,16,17^. Although neuroimaging studies suggest that humans may be neurologically predisposed to interpret dog emotions in similar ways to human emotions^18^, misinterpretations are especially likely when contextual information is lacking^19,20^.

Annotation practices used in these datasets present a further challenge: labels are often created by non-experts, amplifying the risk of potential anthropomorphic bias and misinterpretation^21,22^. While dog owners typically consider themselves confident in interpreting dog emotions, they tend to overestimate their ability, particularly when it comes to negative emotions such as fear^23^. Observer-specific factors such as empathy, cultural background, experience, and attentional focus can significantly affect recognition accuracy^24–26^. Anthropomorphic biases can further complicate interpretation, with humans tending to overemphasise facial cues and neglect body language, whereas dogs themselves rely more heavily on observing the body than facial expressions when observing conspecifics in emotional states^24^.

Given that these human-labelled datasets are increasingly being used to train or evaluate AI systems, such as large vision-language models (LVLMs) like ChatGPT and Gemini, the risk arises that anthropomorphic misinterpretations are not only preserved but amplified at scale. The application of ChatGPT and other LVLMs to tasks for emotion recognition in animals has already begun to emerge. For instance, Cetintav et al.^27^ assessed the performance of GPT-4o on two datasets, the dog emotion dataset and the general animal emotion dataset, and found that while the model achieved 76.6% accuracy on the former, it struggled with the latter, achieving only around 50%. This performance discrepancy underscores the need for a more systematic and critical examination of the application of LVLMs in dog emotion recognition. In the current study, we tested various models on the dog emotions dataset from Cetintav et al., adding newer models with different architectures to their evaluation.

In light of these issues, the emergence of LVLMs presents substantial risks for the task of animal emotion recognition. As these models are trained on massive internet-sourced datasets that include informal media, stock image descriptions, social commentary, and user-generated content, they introduce significant vulnerability to bias. Misconceptions, cultural stereotypes, and anthropocentric assumptions about animal behaviour, and particularly dog emotions, are prevalent in such sources. As a result, these biases may become embedded in the models’ internal representations and influence their outputs, leading to systematic misinterpretation of animal emotional states. Moreover, OpenAI, Google, and Meta have not publicly disclosed the full details of their models’ training data^28^, and based on available studies on the model’s biases towards animals^29^, no steps have been reported towards mitigating anthropocentrism. Just as studies have documented biases in ChatGPT’s and other models’ recognition of human emotions from facial expressions (often linked to factors such as race, age, or gender^30,31^), a similar concern arises when these models are applied to dog emotions. Dogs exhibit a wide range of breed-specific variations in facial morphology, which can significantly affect how expressions are visually perceived, leading to biased classifications across different dog breeds, potentially favouring those more commonly represented in the training data or those whose features more closely resemble anthropomorphic expectations, rather than learning biologically grounded indicators of emotion.

In light of these concerns, the present study aims to critically evaluate the capabilities of state-of-the-art multimodal LVLMs, specifically GPT, Gemini, and LLaVA, in recognising dog emotions from images, uncovering potential biases and limitations. Additionally, this work establishes a benchmark to support future research efforts in the domain of animal emotion recognition using LVLMs. To assess the models’ performance, we conduct a zero-shot evaluation across two datasets: (1) the Dog Emotions (DE)^15^ dataset, consisting of web-sourced images with layperson-generated labels, and (2) the Labrador Retriever (LRc) dataset, derived from videos of dogs in experimentally induced emotional states (positive anticipation and frustration from Bremhorst et al.^8^). We test multiple model variants and prompt types, analyse outputs using standard classification metrics, and explore how contextual features such as artificial background manipulations influence predictions.

## Methods

### Datasets

In the current study, we use two datasets with various dog emotions — the Dog Emotions Dataset and the Labrador Retriever Dataset. Due to the scale of the datasets and the limitations of APIs, we had to limit the number of input images from the original datasets.

***Dog Emotions Dataset (DE).*** The original Dog Emotions Dataset^15^ contains 4,000 images of dogs in four emotional states: *angry*, *happy*, *sad*, and *relaxed* (1,000 each). The dataset description states that images were gathered from various online sources and manually annotated by the author. We randomly selected a subset of 1,000 images (250 of each class) for testing purposes.

***Labrador Retriever Dataset (LR).*** The Labrador Retriever Dataset^8^ contains recordings of 29 *Labrador Retriever* dogs (248 videos total; 19 females and 10 males; age range: 2-9.5 years, mean age $\approx$ 5.22 years) in a controlled laboratory setting, experimentally inducing two emotional states: *positive* (anticipation of a food reward) and *negative* (frustration due to the reward’s inaccessibility). In contrast to the Dog Emotions Dataset, in this dataset, the dogs’ emotions are defined from the experimental context rather than the annotator’s subjective evaluation.

**Fig. 2:**
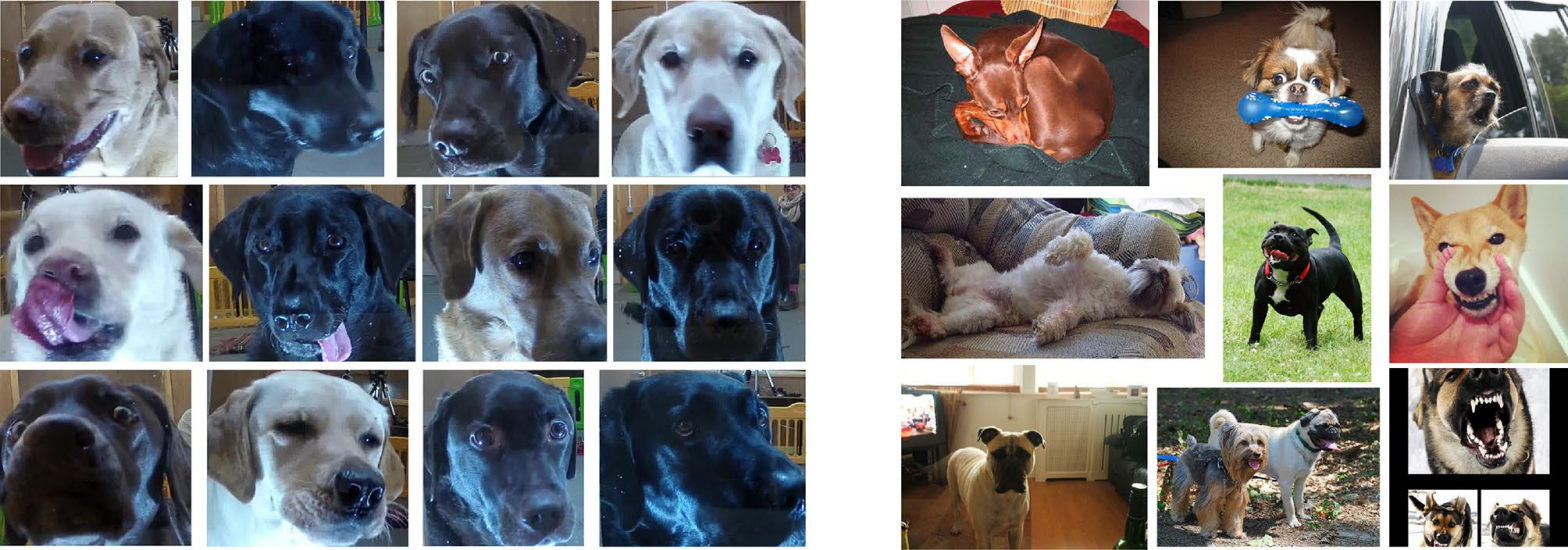
**Example frames from LRc (left) and DE (right) datasets.**.

For image classification, we use the cropped face image dataset (**LRc**), obtained by Boneh-Shitrit et al.^12^. This dataset contains automatically extracted facial images from the frames of videos from the Labrador Retriever Dataset. For the purposes of testing, we randomly selected a subset of 1,000 images (500 of each class). Figure 2 shows images from the LRc and DE datasets.

### Models

To evaluate the performance of large vision-language models in the dog emotion classification task, we selected various popular large language models which can take image data as input. The versions of accessed models are provided in Table 1. All models were tested using Google Colab^32^ and corresponding APIs.

**Table 1:** LVLMs and their versions used in the current study. We are aware that OpenAI’s o4 mini model is technically not a GPT model, but we still refer to it as GPT for consistency.

Model	Version
GPT-4.1^33^	gpt-4.1-2025-04-14
GPT-4.1 mini^33^	gpt-4.1-mini-2025-04-14
GPT-4.1 nano^33^	gpt-4.1-nano-2025-04-14
GPT-o4 mini^34^	o4-mini-2025-04-16
GPT-4o^34^	gpt-4o-2024-08-06
GPT-4o mini^34^	gpt-4o-mini-2024-07-18
GPT-4 turbo^35^	gpt-4-turbo-2024-04-09
LLaVA-NeXT^36^	llava-v1.6-vicuna-7b-hf
Gemini 1.5 Pro^37^	gemini-1.5-pro
Gemini 2.0 Flash^38^	gemini-2.0-flash
Gemini 1.5 Flash^37^	gemini-1.5-flash

### Prompts

To make the results comparable with those of Cetintav et al.^27^, we used the same prompt as in their study, namely “*I will provide you with images of dogs classified into one of N categories: A, B, C ... Your task is to analyse each photo and make a single-word prediction based on these classes. Additionally, explain your reasoning for the prediction by describing the visual cues observed in the image.*”, where *N* is the number of states (*four* in the DE dataset, and *two* in LRc dataset), and *A*, *B*, *C*... — corresponding states (*angry*, *happy*, *sad*, and *relaxed* for the DE; *positive anticipation (positive)* and *frustration (negative)* for the LRc).

Several, especially older models (such as GPT-4 turbo or Gemini 1.5), produced unstructured output, which was almost impossible to parse for the classification task. To structure the outputs and simplify the parsing process, we added an additional prompt after the main one: “*Start with Prediction: and then Reasoning:*”.

We additionally used system prompts to increase the models’ performance^39^. We experimented with several options and selected the “Tree-of-Thought”-inspired^40^ “Panel of Experts”^41^. The underlying idea is that directing LVLMs to operate as a collective of specialists can enhance their problem-solving capabilities and diminish the uncritical acceptance of incorrect initial ideas. Thus, we utilised the following prompt as a system one in all experiments except the prompt variation one: “*Imagine three different dog behaviour experts answering the question. All experts will write down 1 step of their thinking and then share it with the group. Then, all experts will go on to the next step until they are sure. If any expert realises they’re wrong at any point, then they leave*”.

Since we performed a classification task, and generative models occasionally decline to provide an answer (due to internal restrictions or uncertainty), in some models we included a “forcing” prompt: “*You must provide an answer even if you are not sure*.”. This increased the number of meaningful answers, whereas without this prompt, the proportion of classified instances was not suitable for proper result comparison. Models with such an addition are denoted by the $\oplus$ symbol.

We additionally evaluated the performance of the LVLMs when provided a PDF document with the study of Bremhorst et al.^8^, who collected and analysed the original video dataset. The document was attached as an addition to the prompt with the clarification “*The attached PDF contains reference material describing dog facial expressions and emotion classification. Use it to guide your analysis and decision-making.*”.

### Postprocessing and performance evaluation

As generative models are designed to produce text resembling human writing, they occasionally struggle to categorise answers into strictly defined classes, such as emotion categories. To overcome this, we applied response parsing to the outputs provided by the models. First, we filtered the output sequence for the final prediction. If there was one, we compared the subsequent word (or sequence of words) to the ground truth class. To account for synonyms, we defined lists of words that are similar in meaning to the target ones. For example, in the case of the LRc dataset, we categorised answers containing words such as *happy*, *curious*, *excited*, and *relaxed* as belonging to the *positive* class, while words like *frustrated*, *tense*, and *distress* were allocated to the *negative* class. If the prediction differed from all existing options, we assigned the *none* label to the corresponding answer; otherwise, we assigned the label of the matched class.

Then, if there was no final prediction, we filtered the answer for keywords that corresponded to the model’s refusal to respond, such as *unable*, *can’t classify*, *not sure*, *uncertain*, and so on. If any were present, we assigned the *none* label. Additionally, we examined whether the answer included words from more than one class synonyms list, as sometimes models do not directly answer the question, opting instead to provide descriptive information (such as “*Look for signs of relaxation or tension in the dog’s posture. A relaxed dog might have a loose body and a wagging tail, while a tense dog may present a stiff posture or a lowered tail”*). If there were only words related to one class, we assigned the label of that class. Finally, if the answer contained none of these words, we assigned it a *none* label. Filtered answers obtained during the parsing step were subsequently compared with the ground truth labels using standard classification metrics (accuracy, F1 score, and confusion matrix). It is essential to note that, as the predictions included the *none* class, we excluded it from the performance evaluation process, focusing solely on classified instances. To account for this, we also assessed the proportion of unclassified instances in each case (the number of classified images divided by the total number of images).

To move beyond purely descriptive metrics, we formally assessed model performance using inferential statistics. For within-dataset comparisons (e.g., among GPT variants on DE), we computed McNemar’s exact test on per-sample correctness to evaluate whether differences in accuracy between models were statistically significant. In parallel, we applied non-parametric bootstrap resampling (1,000 replicates) to estimate 95% confidence intervals for the differences in accuracy and F1 scores between model pairs, providing robust effect size estimates that do not rely on normality assumptions. For cross-dataset generalisation (DE $\xrightarrow {}$ LRc), we repeated the bootstrap analysis within each model to quantify the magnitude and statistical reliability of performance drops, thereby testing whether performance changes were attributable to sampling variability or reflected genuine failures to generalise.

### Background manipulation

**Fig. 3:**
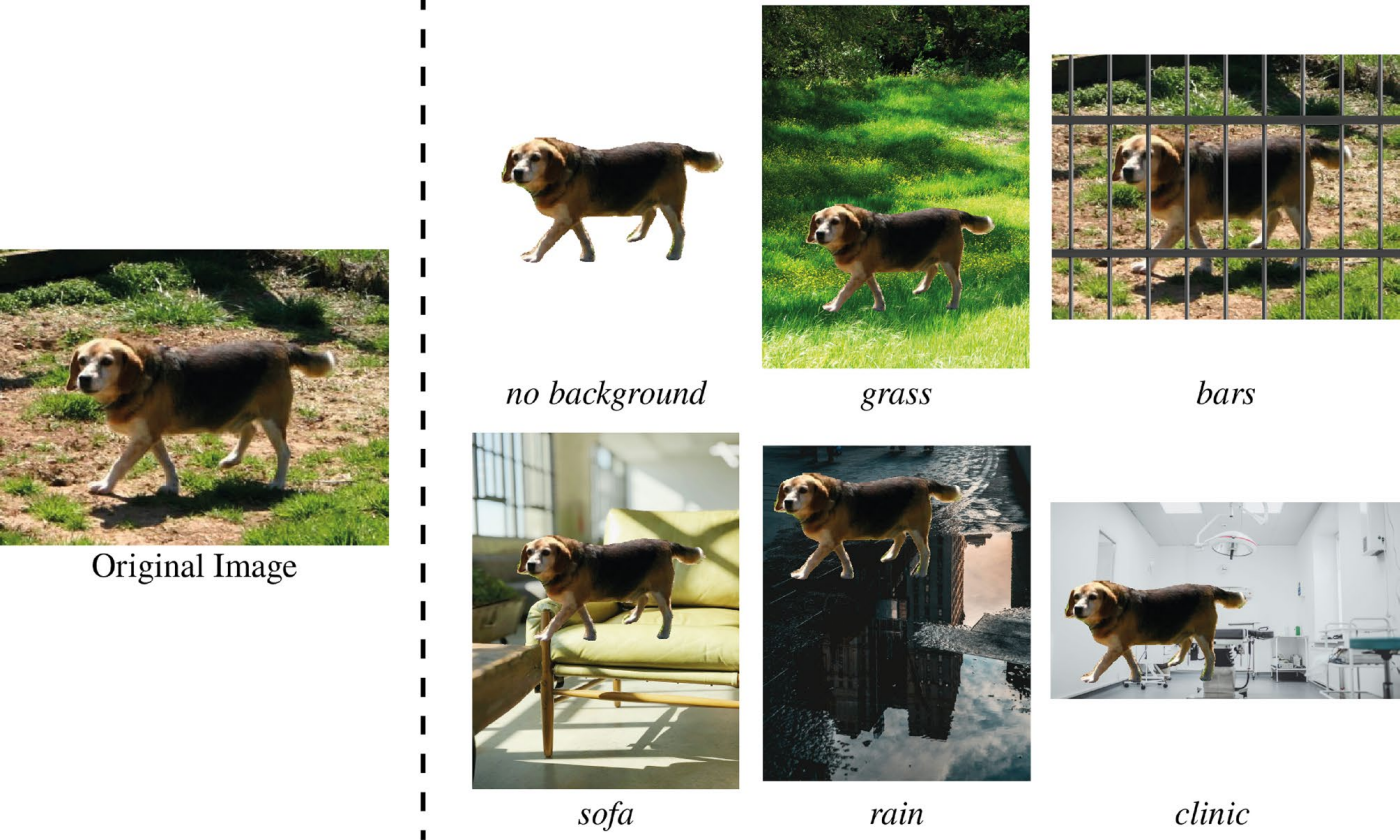
Original image from the Dog Emotions (DE) dataset and processed images, containing a segmented dog image placed on various backgrounds. Original image is from the DE dataset^15^. The grass image by @lisaleo, sofa image by @cortes, rain image by @dancalders, and clinic image by @tetrakiss are all from Unsplash. Bars image by upklyak from Freepic.

To assess the impact of the image background on classification accuracy, we preprocessed images from the DE dataset, separating dogs from the background with the YOLOv11 segmentation model^42^, pretrained on the COCO dataset^43^. We then placed dog images onto selected backgrounds in random places, adjusting their sizes by the background size. We processed all images from the DE dataset, retaining the segmented dog images (*no background*) and placing the dogs onto a variety of backgrounds (same set for each image), including *grass*, *sofa*, *rain*, and *clinic*. Additionally, we overlaid an image of metal bars with a transparent background onto the original images, resulting in *bars* image class. Examples of an image with altered backgrounds are shown in Fig. 3.

## Results

### Evaluation on the dog emotions (DE) dataset

**Fig. 4:**
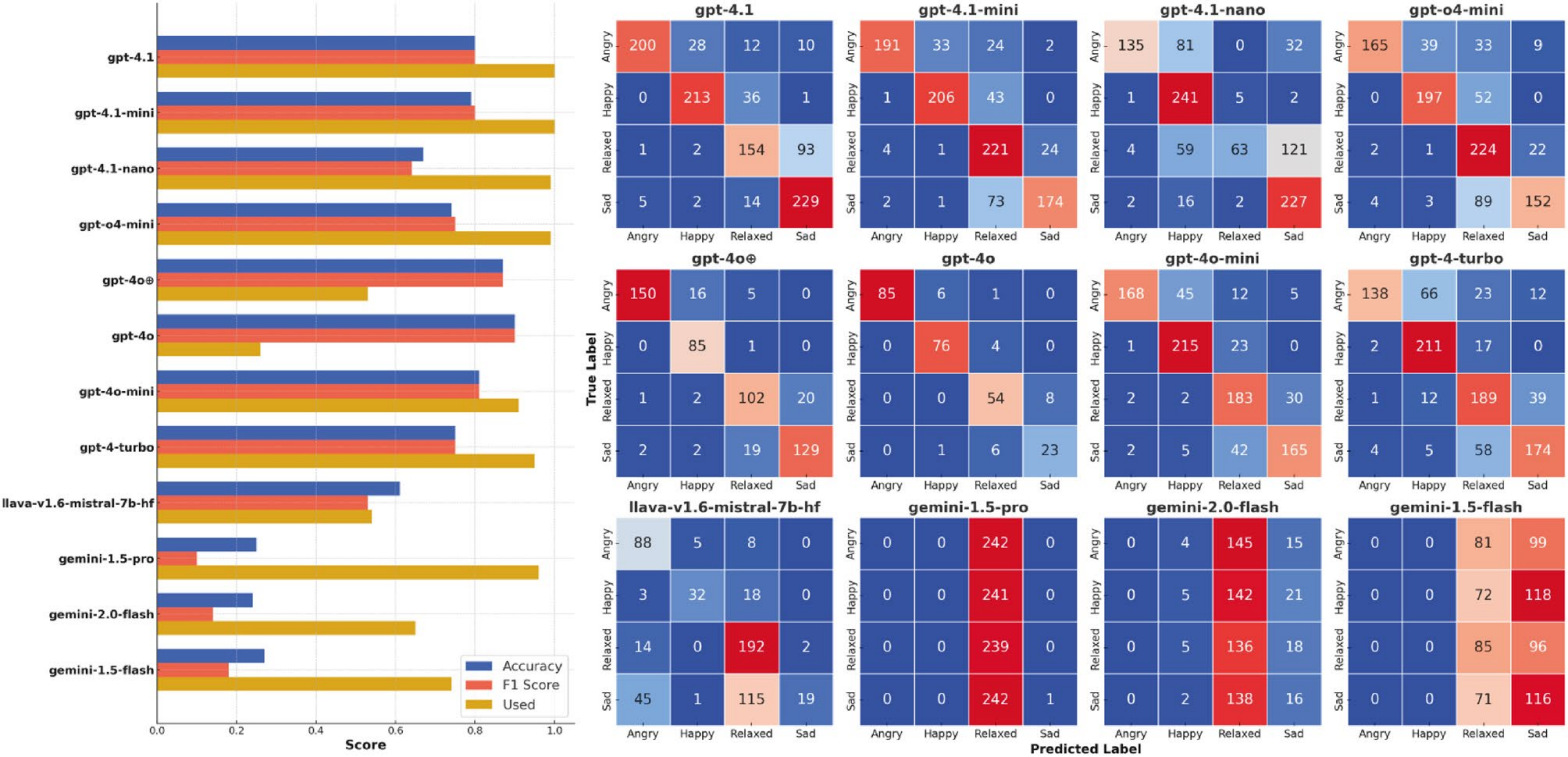
Performance on the DE dataset: Left. Models’ accuracy, F1 score and portion of the images used (with a class assigned). Right. Confusion matrices for the classification.

First, we investigated the performance of various LVLMs on the Dog Emotions (DE) dataset^15^. Taking into account the lack of validity of the labels in this dataset and reliability in the annotation, we provide classification results to establish a baseline for subsequent experiments and compare them to the results of Cetintav et al.^27^, who investigated the performance of a GPT-4o model on the same dataset. Since we did not have access to the exact filtered subset used in the original study, the straightforward accuracy comparison is impossible, but the provided results (Fig. 4 left) demonstrate a comparable level of performance for the GPT models, with Gemini models performing significantly worse. It is worth noting that more advanced models (such as GPT-4o or Genimi 2.0) tend to “refuse” to classify more images than their less advanced versions, which could be connected with models’ uncertainty or more advanced reasoning and leads to the reduced number of images classified. This, however, is not true for the more recent GPT 4.1 series. Confusion matrices (Fig. 4 right) demonstrate mixed results, with no significant “favouritism” for GPT models and strong bias for LLaVA and Gemini models, which exhibit a prediction collapse, consistently outputting *relaxed* and *sad* classes. Inferential analyses confirmed these trends: GPT-4o variations significantly outperformed smaller GPT-4.1-nano, LlaVA, and all Gemini models (McNemar $p < 0.001$, bootstrap $\Delta$Acc 0.08–0.15, 95% CIs excluding 0), while GPT-4.1 and GPT-4.1-mini were statistically indistinguishable (McNemar $p > 0.1$).

**Fig. 5:**
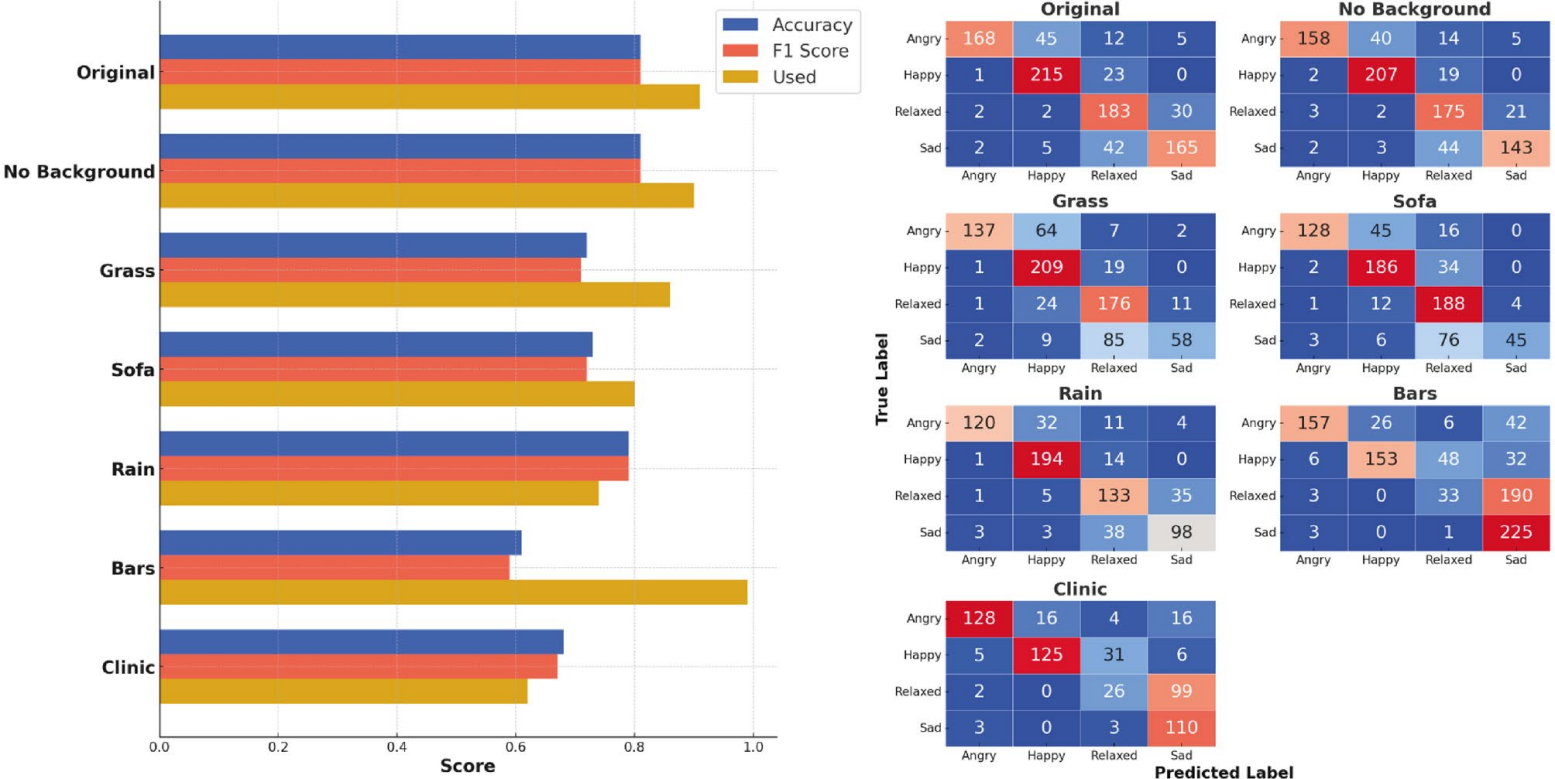
Performance of GPT-4o mini model on the DE dataset with various artificial backgrounds: Left. Model’s accuracy, F1 score and portion of the images used (with a class assigned). Right. Confusion matrices for the classification.

Having established the baseline, we selected the GPT-4o mini model to investigate the influence of the background on emotion classification, and, therefore, determine if the model “pays attention” to the visual appearance of the dog itself, rather than to the surroundings. The results of the experiment (shown in Fig. 5) demonstrate that the background indeed affected the model’s performance, influencing the classification results in the way the background implies. For instance, images with *grass* and *sofa* backgrounds tend to be classified less as *sad* and more as *happy* and *relaxed*. On the opposite side, images with *clinic* background and *bars* overlaid were misclassified as *sad*, almost negating the *relaxed* class. Images with the *rain* background, surprisingly, were the most misclassified between *relaxed* and *sad* classes. The proportion of non-classified images increased in all classes with a manipulated background, except for the *bars* class. This may be caused by the “obvious” artificiality of some images, where the segmented dog is cropped, or does not correlate in size with its environment, which potentially leads to confusion and “refusal” to assign a label. Moreover, we did not observe a significant difference in performance between the original images and images with no background.

### Evaluation on the Labrador Retriever (LRc) dataset

**Fig. 6:**
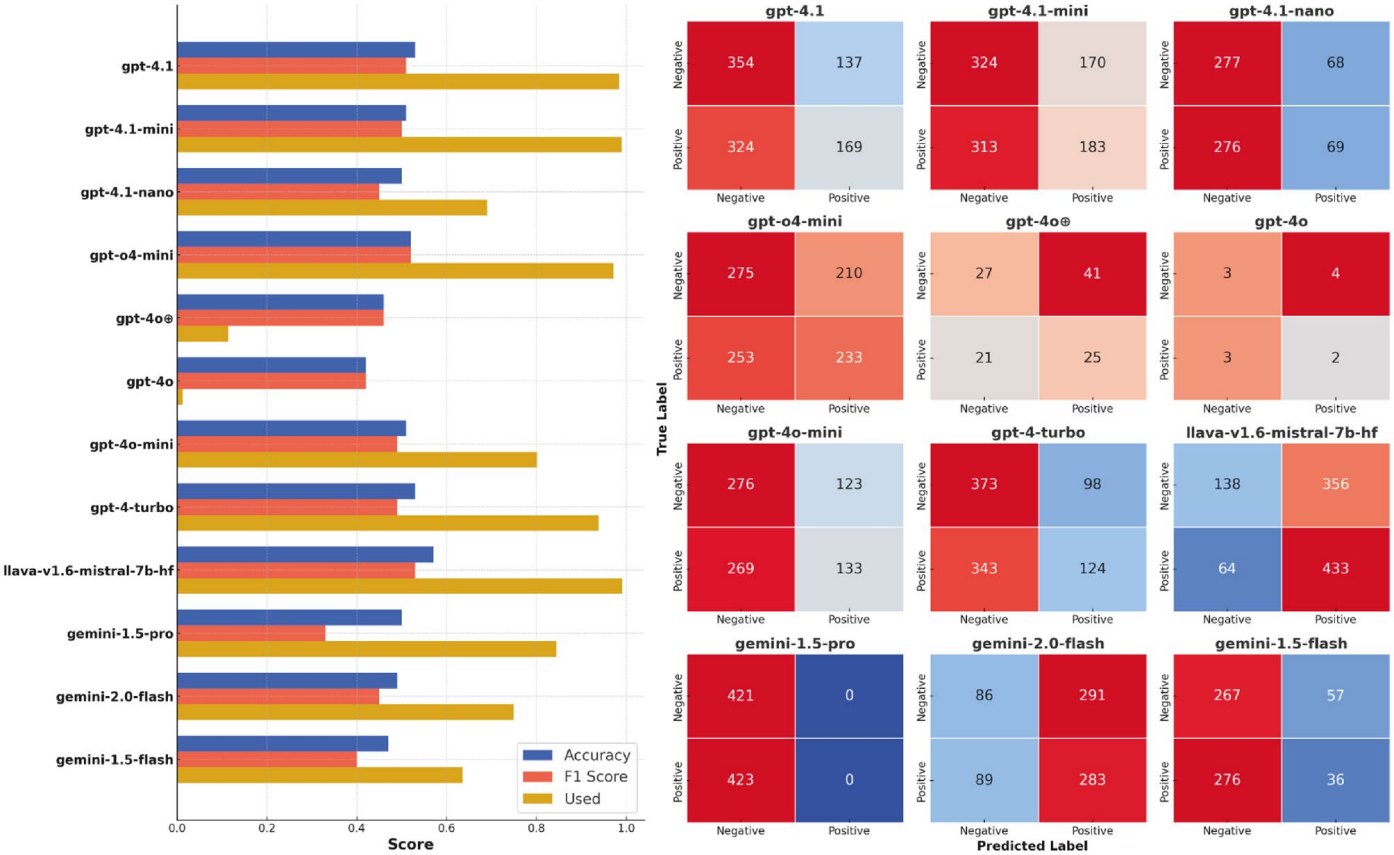
Performance on the LRc dataset: Left. Models’ accuracy, F1 score and portion of the images classified. None of the models achieved an accuracy greater than 0.6, performing close to the chance level. Right. Confusion matrices demonstrate that most of the models tend to attribute one class to almost all images.

The Labrador Retriever dataset (LRc) contained cropped faces with minimal background and emotions implied by experimental context. This approach ensured that the model made decisions based solely on the dog’s face, and its decision could be compared to a more objective label. The classification results, shown in Fig. 6, demonstrate three key changes in comparison to the performance on the DE dataset. First, the general performance dropped significantly, with all models performing close to the chance level. Bootstrap 95% confidence intervals for these differences excluded zero ($\Delta$Acc $\approx$ -0.32 to -0.55), and McNemar’s tests confirmed the drops as highly significant for all models ($p < 0.001$). Second, all models (except for GPT-o4 mini) demonstrate collapsed predictions — a strong preference towards one class (not always positive, though). Third, the number of classified images dropped in the majority of models. The most accurate in the first experiment, the GPT-4o model classified only 12 out of 1,000 images, and 114 when forced by the prompt. Overall, the results demonstrate much worse performance of LVLMs on the LRc dataset.

We further experimented with system prompts to potentially improve the performance of the GPT-4o mini model on the LRc dataset. The results shown in Table 2 demonstrate no significant difference in performance with various prompts.

**Table 2:** The accuracy of GPT-4o mini model predictions on the LRc dataset with different system prompts. *Panel of Experts* denotes the prompt used in the rest of the experiments (see the details in the Methods section). *Study* denotes the PDF file of the study of Bremhorst et al.^8^.

System Prompt	Accuracy	F1
*None*	0.48	0.44
You are a dog	0.48	0.46
You are a dog behavioural expert	0.49	0.46
*Panel of Experts*	0.51	0.49
*Panel of Experts* + *Study*	0.51	0.5

## Discussion

Recognising emotional states in animals is a scientifically complex and ethically important task, with far-reaching implications for animal welfare and human-animal relationships. This task is also inherently difficult due to the absence of verbal self-report in non-human species and the limitations of current behavioural and physiological indicators used as proxies.

AI algorithms for emotion recognition have become well-established in the human domain and are now increasingly being applied to animals. However, since these systems are trained on data labelled by humans, they inevitably inherit and often amplify the anthropomorphic assumptions and cultural biases embedded in that data. Consequently, their interpretations of animal emotions may reflect human-centred perspectives rather than objective, biologically grounded assessments. This raises critical concerns about the validity and reliability of such models, particularly when used in scientific, veterinary, or welfare contexts where an accurate understanding of animal emotional states is essential. The use of large vision-language models (LVLMs) amplifies these concerns due to their training on large uncurated datasets, which have already been demonstrated to have various kinds of biases in the human domain, such as gender^44–46^, cultural background^47,48^, disability^49^, and many others^50^.

The present study is the first to systematically investigate the use of LVLMs for recognising emotions in dogs from different datasets. To establish a baseline and examine potential biases, we began by replicating the experimental setup of Cetintav et al.^27^ using the Dog Emotions dataset, yielding comparable performance metrics. We then probed the model’s sensitivity to contextual features by systematically altering the backgrounds of the images while keeping the dog’s appearance unchanged. The results revealed a significant shift in emotion classification outcomes depending on the background context. Specifically, images featuring naturalistic or domestic settings, such as a grassy field or a sofa, were more frequently classified as conveying positive emotional states like “happy” or “relaxed”. In contrast, the same dog placed in clinical or confined environments, including a veterinary setting or cage-like bars, was more often classified as expressing negative emotions. These findings suggest that LVLMs do not primarily rely on intrinsic features of the dog, such as facial expressions, posture or other behaviourally relevant features, when inferring emotional states. Instead, their predictions appear to be strongly influenced by extraneous visual cues in the background.

To address this issue further, the second step of our experiments focused on the LRc dataset, which includes cropped facial images with minimal background from frames of original videos collected by Bremhorst et al.^8^. Remarkably, on this dataset, the model’s performance dropped to near-chance levels. In contrast to the DE dataset, where layperson-generated labels may reflect anthropomorphic assumptions that the models learnt during training, the LRc dataset provides more scientifically grounded labels based on carefully controlled emotion elicitation procedures. This dramatic drop in classification accuracy across all models confirms that LVLMs are currently not effectively leveraging biologically relevant facial cues in the task of emotion classification. While confidently labelling dogs pictured as “happy”, the models struggled to classify cropped dogs based solely on their faces. Importantly, the poor zero-shot performance is not due to an absence of emotional information in the facial images themselves. Boneh-Shitrit et al.^12^ demonstrated that a deep learning model based on a DINO-ViT backbone can achieve 89% accuracy on the same cropped dog face dataset, indicating that informative facial signals are indeed present, but not effectively leveraged by current zero-shot LVLMs. It is important to emphasise that in the DE dataset, models can potentially focus on various behavioural cues beyond just facial expressions, such as posture and context. This can improve their performance; however, it has been demonstrated that they may rely on these cues too heavily.

Interestingly, newer models, such as GPT-4o, often refused to classify images. While this refusal to answer may reflect improved calibration or uncertainty handling, it may also hint at limitations in model confidence when operating in the domain of dog emotions. Given that prompt phrasing can significantly influence the outputs of large language and vision-language models^51^, an important direction for future work is systematic exploration of prompt design and variation. This includes testing whether more structured, specific, or contextually informative prompts can improve performance. Yet it should be noted that to address the low response rate, we did experiment with various prompt engineering strategies, including multi-agent setups such as the “Panel of Experts” approach, and system-level directives encouraging classification even under uncertainty. While these strategies modestly increased the number of responses generated, they did not lead to any meaningful improvement in classification accuracy, which is consistent with prior work showing that expert-style prompting reduces refusal rates by distributing uncertainty across simulated “agents”, but maintaining errors and biases highly correlated between the instantiations of the same model^52^. This suggests that surface-level prompt manipulation alone is insufficient to resolve the deeper challenges involved in extracting biologically relevant insights from vision-language models in this context. 

Another promising direction for future research involves more sophisticated experimentation with background manipulation. In the current study, we conducted only a basic form of background replacement, without adapting factors such as lighting, perspective, or the relative scale of the dog to match the new environment. As a result, many of the generated images appeared visually unnatural. This issue was further compounded by the fact that a significant portion of the dataset included dogs that were only partially visible: when these segmented figures were randomly placed onto new backgrounds, the resulting compositions often appeared even more artificial or fragmented. Interestingly, these visual inconsistencies did not have a major impact on model performance (aside from reducing the number of images the models were willing to classify), suggesting a notable limitation in the models’ spatial reasoning and contextual integration. In particular, the models seemed insensitive to the implausibility of the visual scene, reinforcing earlier findings that current vision-language models often lack robust spatial awareness^53,54^. This insensitivity raises questions about the depth of the models’ visual understanding and their reliance on texture, colour, or object presence rather than coherent scene interpretation. Future work should explore more ecologically valid methods of background editing to better assess how these models process spatial and contextual cues.

Few-shot learning and fine-tuning could potentially greatly improve LVLMs’ ability to perform animal-related tasks and reduce internal biases. As shown previously for various models^55–58^, introducing even a small amount of data may lead to better accuracy. The quality of the data is, of course, crucial. In future studies, we plan to utilise few-shot learning and fine-tuning to investigate how such techniques affect the performance of LVLMs on different datasets.

Our findings demonstrate that current LVLMs are not yet suitable for reliable, biologically grounded recognition of canine emotions. Although some models showed moderate performance on the Dog Emotions and similar datasets, these results may be misleading due to the models’ reliance on contextual elements, such as background or scene composition. This poses significant risks if such models are integrated into real-world applications: in veterinary contexts, on-farm welfare monitoring, or others. Even in commercial products aimed at pet owners, misleading emotional feedback could foster false confidence in the technology and skew human-animal interactions. As the use of LVLMs becomes more widespread, ensuring that their inferences are grounded in scientifically validated behavioural indicators rather than superficial visual patterns is both an ethical and practical imperative. This has already begun to happen in the field of bioacoustics, where NatureLM-audio, the first audio-language foundation model specifically designed for bioacoustics and trained on a carefully curated bioacoustic dataset, was recently introduced^59^. We hope that the current study contributes to the scientific discourse on promoting an analogous paradigm shift in the domain of AI-powered animal emotion recognition using the visual modality.

## Data Availability

The datasets used in this study are parts of previously published datasets (8, 15). The exact images used for testing are available here (https://drive.google.com/drive/folders/1uo47ZTJiIKghIlTwQvhueGOKyJwcmrkC?usp=drive_link). The code generated during this study is available from the corresponding author upon reasonable request.
